# Letter to the Editor: The Lineage and Sublineage Investigation of Human Papillomavirus Type 16 in Tehran, Iran, During 2022–2023

**DOI:** 10.1002/hsr2.71260

**Published:** 2025-10-02

**Authors:** Shanza Shakir, Syed Hassan Ali, Javed Iqbal

**Affiliations:** ^1^ Liaquat University of Medical and Health Sciences (LUMHS) Jamshoro Sindh Pakistan; ^2^ ⁠Nursing Department Hamad Medical Corporation Doha Qatar

Dear Editor,

We have read the article published by Khezeli et al., titled *“The Lineage and Sublineage Investigation of Human Papillomavirus Type 16 in Tehran, Iran, During 2022–2023: A Cross‐Sectional Study”* (Health Science Reports, 2025). In this study, he highlights the geographic diversity of distinct HPV 16 sublineages worldwide and found that lineages A and D were dominant in Tehran, Iran. However, there are some limitations that we want to emphasize to further magnify this novel topic; Firstly, this study included only 120 participants, which made the study's sample size small, and all data were collected from participants in Tehran only. The study's small sample size may raise the margin of random error and reduce the study's statistical power, due to which it limits the ability to find significant relationships. Additionally, collecting data from a specific geographic area limits the generalizability and leads to selection bias in the main findings for the entire Iranian population. A study conducted by Siavash Chalabiani et al., collected HPV type 16 data from 2,969 women across 24 provinces in Iran [1]. The significant number of participants and geographically varied sample of this study ensures greater accuracy and lesser uncertainty; improve statistical power and reduce selection bias, which strengthens the generalizability and reliability of the findings. Secondly, this study focuses only on E6 gene analysis, which provides limited insights into the genetic diversity of this virus. Whole genome sequencing (WGS) is the more effective method to evaluate the full spectrum of HPV 16 sublineage diversity. As evidenced by studies that located several variations in HPV 16 genomes from various populations, WGS enables to find the wider variety of genetic variants throughout the entire genome, including single‐nucleotide polymorphisms (SNPs) and insertions/deletions (Indels) [2]. WGS, including E6, E7, E1, E2, and the long control region (LCR) is necessary for precise lineage categorization since the behavior of the virus and its correlation with cervical cancer can be greatly impacted by mutations in all these areas. Cervical cancer may arise as a result of the integration and mutation of E1 and E2, which may increase the expression of virus genes E6 and E7. The E1 gene is involved in the replication of the viral genome and the E2 gene has a negative impact with the production of oncogenes. The E6 and E7 genes are primarily responsible for HPV's ability to cause cervical cancer [3].

Additionally, this study would not be able to differentiate between D1 and D4 sublineage, using E6 gene analysis only, results in a gap in phylogenetic resolution. A single gene analysis might not be able to fully reflect the complexity of genetic variants, unlike the phylogenetic analyses, which were carried out with MAUVE. MAUVE phylogenetic analysis evaluates the genetic composition of sublineages like D1 and D4. MAUVE phylogenetic analysis helps researchers to compare genomic sequences in great detail and to find particular mutations and changes within these D1 and D2 sub lineages that might be linked to an elevated risk of cancer in these particular sublineages [4].

Lastly, this study does not include host genetic data, for example, HLA typing which limits the association between viral variants with host susceptibility. Cervical cancer risk is influenced by HLA gene variation, which has been connected to varying immunological responses to HPV [5]. A complicated interaction between host genetics and viral components is indicated by the correlation between specific HLA haplotypes and cytokine levels, which may impact the course of the disease [5].

In conclusion, we want to thank the author for giving us the privilege to further investigate this remarkable topic to enhance the findings regarding the limitations. To further amplify this topic, future research should include larger and geographically diverse populations, use WGS, and integrate host genetic factors like HLA typing to improve lineage classification, evaluate virus‐host interactions, and guide towards targeted prevention and early diagnosis strategies for cervical cancer.

## Author Contributions

**Shanza Shakir:** conceptualization, data curation, formal analysis, resources, writing – original draft, and writing – review and editing. **Syed Hassan Ali:** conceptualization, supervision, data curation, formal analysis, resources, writing – original draft, and writing – review and editing. **Javed Iqbal:** writing – original draft, writing – review and editing.

## Ethics Statement

The authors have nothing to report.

## Consent

The authors have nothing to report.

## Conflicts of Interest

The authors declare no conflicts of interest.

## Data Availability

The authors have nothing to report.
